# Plant-Produced Vaccines: Future Applications in Aquaculture

**DOI:** 10.3389/fpls.2021.718775

**Published:** 2021-08-12

**Authors:** Hang Su, Igor A. Yakovlev, André van Eerde, Jianguo Su, Jihong Liu Clarke

**Affiliations:** ^1^Division of Biotechnology and Plant Health, NIBIO - Norwegian Institute of Bioeconomy Research, Ås, Norway; ^2^Department of Aquatic Animal Medicine, College of Fisheries, Huazhong Agricultural University, Wuhan, China

**Keywords:** plant genetic engineering, aquaculture, fish vaccine, food security, application prospects

## Abstract

Aquaculture has undergone rapid development in the past decades. It provides a large part of high-quality protein food for humans, and thus, a sustainable aquaculture industry is of great importance for the worldwide food supply and economy. Along with the quick expansion of aquaculture, the high fish densities employed in fish farming increase the risks of outbreaks of a variety of aquatic diseases. Such diseases not only cause huge economic losses, but also lead to ecological hazards in terms of pathogen spread to marine ecosystems causing infection of wild fish and polluting the environment. Thus, fish health is essential for the aquaculture industry to be environmentally sustainable and a prerequisite for intensive aquaculture production globally. The wide use of antibiotics and drug residues has caused intensive pollution along with risks for food safety and increasing antimicrobial resistance. Vaccination is the most effective and environmentally friendly approach to battle infectious diseases in aquaculture with minimal ecological impact and is applicable to most species of farmed fish. However, there are only 34 fish vaccines commercially available globally to date, showing the urgent need for further development of fish vaccines to manage fish health and ensure food safety. Plant genetic engineering has been utilized to produce genetically modified crops with desirable characteristics and has also been used for vaccine production, with several advantages including cost-effectiveness, safety when compared with live virus vaccines, and plants being capable of carrying out posttranslational modifications that are similar to naturally occurring systems. So far, plant-derived vaccines, antibodies, and therapeutic proteins have been produced for human and animal health. However, the development of plant-made vaccines for animals, especially fish, is still lagging behind the development of human vaccines. The present review summarizes the development of fish vaccines currently utilized and the suitability of the plant-production platform for fish vaccine and then addresses considerations regarding fish vaccine production in plants. Developing fish vaccines by way of plant biotechnology are significant for the aquaculture industry, fish health management, food safety, and human health.

## Introduction

Aquatic products including fishes, crustaceans, and other miscellaneous aquatic animals are excellent animal protein sources and contain a great variety of nutrients which are essential for humans (Adams, 2019). Among these species, fishes and fish source products are the most consumed. Fisheries also play an important role in the livelihoods of millions of people worldwide. Currently, the most important methods to obtain fishes or other aquatic products are capture from seawater or freshwater and aquaculture. Fish, crustaceans, mollusks, and aquatic plants can all be cultured in aquaculture industries; of these, fish farming is the most important (Evensen, 2016).

The global fishery production (capture and aquaculture) reached 206.5 million tons in 2017 and increased to 211.9 million tons in 2018. Aquaculture has expanded impressively in the past several decades and has become the major global fish industry with over 50% of the total fishery production with 112.2 million tons in 2017, increasing to 114.5 million tons in 2018 (FAO, 2021). Production of aquaculture finfish (including marine fishes, diadromous fishes, and freshwater fishes) constituted a large part of the total production, amounting to 52.7 million tons in 2017 and 54.3 million tons in 2018 (FAO, 2021).^1^ Aquatic products are mostly from Asia, in particular China, which covers over 70% of global aquaculture production with a great increase (99.6 million tons in 2014–120.1 million tons in 2019, FAO, 2021; see footnote 1) in these years. It is estimated that in this decade, total production from both capture and aquaculture will exceed that of beef, pork, or poultry due to the high global demand for food fish (Clarke et al., 2013). The rapid development of aquaculture could contribute to the UN sustainable development goals [SDGs, especially SDGs 1 (no poverty), 2 (zero hunger), 3 (good health and wellbeing), 14 (life under water), and 15 (life on land)].^2^

Since the rapid advance of aquaculture, the high densities and other artificial conditions (artificial propagation, polyculture, transportation etc.) in which fish are farmed exponentially increase the risks of outbreaks from infectious diseases. There may also be an ecological hazard in terms of pathogen spread to wild fish (Visuthismajarn et al., 2005). It is estimated that approximately 10% of cultured aquatic animal production are lost due to infectious diseases globally, leading to over 10 billion USD economic waste (Adams, 2019). Outbreaks of diseases in Atlantic salmon (*Salmo salar*), oyster and marine shrimp have led to partial or sometimes total loss of production in several countries worldwide. Natural disasters, diseases, and pollution have together caused serious production loss globally (Su et al., 2021).

Aquatic diseases also threaten human livelihood and health by reducing food security. Unhealthy aquatic production and drug residues from production may have harmful effects on humans and the environment. People increasingly pay attention to the quality and safety of aquatic products and the risk to these from environmental pollution. To control disease occurrence, chemicals and antibiotics are employed. Antibiotics, such as florfenicol, norfloxacin, and flavomycin, can protect farmed fish from bacterial diseases efficiently and cost-effectively. However, these may pose serious risks to the environment, human health, and food security (Sneeringer et al., 2019). Drug residues with the potential risk of drug resistance, allergic reactions, and poisoning reactions seriously endanger the farmed species, the environment, and human health, for which the emerging antimicrobial resistance (AMR) has become a global threat (Svoboda, 2020). Therefore, health management of aquaculture is of great importance for food security, food safety, the environment, and sustainable development of the aquaculture industry. Moreover, candidate alternatives, e.g., probiotics and plant bioactive compounds to replace antibiotics, could also be promising, contributing to the management of aquaculture fish health (Yao et al., 2020).

To secure the sustainability and expansion of global aquaculture production, fish vaccination has been proven highly effective and safe, protecting fish from diseases with minimal ecological impact and being applicable to all species of farmed fishes. Vaccination is the mainstream technology for fish disease prophylaxis worldwide and the normative production standard for international modern aquaculture. Since the 1980s, development of fish vaccines has made significant progress. The number of commercial vaccines available for use in fish expanded from two in the 1980s to 34 currently (Shefat, 2018). Up to 2020, over 140 fish vaccines have received approval globally, according to incomplete statistics (Jeong et al., 2020). Vaccine types mainly include live vaccines, inactivated vaccines, and genetically engineered vaccines. Recombinant subunit vaccines, one kind of genetically engineered vaccine, represent promising options for high safety, high stability, easy production, easy control, and good immunogenicity (Su and Su, 2018). Subunit antigens have been produced in bacteria, yeast, transgenic plants, insects, mammalian cell cultures, and cell-free platforms.

In the present review, we summarize the development status of fish vaccines and describe the potential of plant biotechnological engineering for their further development.

## Fish Vaccine Development

Intensive and large-scale fish farming has created conditions in which rapid spread and vast outbreaks of all kinds of infectious fish diseases can occur (Adams, 2019). Due to the challenges in development of vaccines, a large number of drugs, especially antibiotics, have been widely used in fish farms to improve fish growth and enhance productivity (Adams, 2019). However, the utilization of antibiotics in aquaculture results in drug residues, leading to environmental pollution seriously threatening not only ecological safety but also human health. Additionally, it exacerbates the global AMR threat (Svoboda, 2020).

Fish have an integrated immune system including an innate immune system and an adaptive immune system. Both innate and adaptive immune systems are essential for vaccination to trigger an immune response. In 1942, inactivated *Aeromonas salmonicida* orally immunized rainbow trout (*Oncorhynchus mykiss*) as the first fish vaccine (Duff, 1942). Researchers have since made extensive studies to prepare different kinds of vaccines to control fish diseases caused by virus, bacteria, or parasites. Large numbers of vaccines have been reported for Atlantic salmon, rainbow trout, sea bass (*Dicentrarchus labrax*), sea bream (*Sparus aurata*), tilapia (*Oreochromis niloticus/mossambicus*), amberjack (*Seriola dumerili*), yellowtail (*Seriola quinqueradiata*), catfish (*Ictalurus punctatus*), and Vietnamese catfish (*Pangasianodon hypophthalmus*; Clarke et al., 2013; Assefa and Abunna, 2018). There are currently over 30 commercially available vaccines against major infectious bacterial and viral diseases of fish, including *Arthrobacter* vaccine, *Vibrio anguillarum-ordalii*, *A. salmonicida* bacterin, *Yersinia ruckeri* bacterin, and other vaccines against bacteria in salmonids, *Flavobacterium columnare* vaccine, and *E. ictaluri* bacterin against bacteria in grouper, infectious pancreatic necrosis virus (IPNV) vaccine, infectious salmon anemia vaccine, nodavirus vaccine, and other vaccines against viruses in salmonids and seabass, *Streptococcus agalactiae* vaccine, and *Streptococcus iniae* vaccine against tilapia Streptococcosis, as well as spring viremia of carp vaccine, koi herpes virus (KHV) vaccine, grass carp hemorrhage disease vaccine, and other vaccines against viruses in carps (Shefat, 2018).

Fish vaccines are normally classified into three types based on preparation methods; these are live vaccine, inactivated vaccine, and genetically engineered vaccine (Table 1). Live vaccines are prepared with pathogens managed by attenuation or mutated attenuation. Inactivated vaccines are inactivated pathogenic microorganisms that remain immunogenic. They have the ability to induce specific resistance in aquatic animals after inoculation. For genetically engineered vaccines, different types include recombinant subunit vaccines, DNA vaccines, gene deletion/mutant vaccines, and live-vector vaccines. The most widely applied fish vaccines currently are inactivated and live attenuated vaccines (Ma et al., 2019), for example, KHV inactivated vaccine, SVCV inactivated vaccine, carp herpes virus live vaccine, and GCRV live vaccine (Su and Su, 2018). Both types have some disadvantages. Inactivated vaccines require large numbers of antigens with high cost, while attenuated live vaccines are associated with long-term biosafety concerns from the risk of reversion to virulence and also stability issues due to their sensitivity to temperature and light (Su and Su, 2018).

**Table 1:** Comparison of three types of vaccines and delivery methods.

	Live vaccine	Inactivated vaccine	Subunit vaccine
Preparation method	Attenuation/mutated attenuation	Inactivated	Recombinant
Common delivery method	Injection/immersion	Injection/immersion	Injection/immersion/oral administration
Administration ease	Laborious	Laborious	Convenient for oral administration
Immunity duration	6–12 months	6–12 months	Depends on delivery method
Cost	High cost	High cost	Cost-effective
Advantages	Generally attenuated vaccine; Close to natural infection; Effectively stimulates immune system; Pathogen can reproduce *in vivo*; Low dosage; No adjuvant needed; and Long protection duration	Short development cycle; Safe to use; and Easy to preserve	Excellent safety; Simple production; Easy control; High stability; High purity and Good immunogenicity; The most promising vaccine and Low-cost Important direction of vaccine development
Limitations	Inconvenient storage and transportation;Poor safety under natural conditions; and Short shelf life	Cannot reproduce after immunization; Large immunization dosage; Short duration; and Appropriate adjuvant is needed	Highly affected by the expression system; Uncertain immune response caused by the vaccine; Tissue distribution and expression still unknown; Uncertain stability; Immune tolerance; Short protection duration; and Not many successful cases

Delivery methods for fish vaccines include injection, immersion, and oral administration. Intramuscular or intraperitoneal injection is the most popular method, providing an exact vaccination dosage and regulating systemic immune responses, but it usually causes large fish stress responses and is time-consuming, laborious, and relatively expensive (Corbeil et al., 2000). For immersion, the mechanism of fish vaccination during immersion immunization is still uncertain. However, vaccine concentration, immersion time, aquatic animal size, adjuvants, antigenic forms, and water temperature are all factors affecting the uptake of the soaked immune antigens by the hosts (Su and Su, 2018).

Since aquatic animals live in an environment inseparable from water and are cultured in large bodies of water, oral immunization is considered to be the most ideal vaccination method for fish and other aquaculture animals. Oral vaccination provides a non-stressful and energy-saving administration for both fish and farmers (Embregts and Forlenza, 2016). However, oral vaccination procedures are difficult to standardize. The large surface area of the intestine increases the possibility of antigen breakdown in the gastro-intestinal tract, leading to mostly local mucosal immune responses and immune tolerance. Vaccination dosage, vaccination regime, antigen formulation, encapsulation, adjuvants, and the identity of the fish species are all necessary factors to be considered for oral administration, and further research is needed to achieve a balance between immune responses and tolerance (Embregts et al., 2019). Oral vaccination along with commercial feed of *Escherichia coli* derived orange-spotted grouper nervous necrosis virus (NNV) virus-like particle (VLP) vaccine was able to offer over 50% survival against NNV infection (Chien et al., 2018). Lyophilized recombinant yeast producing red grouper nervous necrosis virus capsid protein as diet for groupers is reported to be feasible for vaccination (Cho et al., 2017).

Each kind of vaccine has its own advantages and shortcomings, and the choice of vaccine and delivery method is largely dependent on characteristics of the farmed fish species, such as size, feeding habits, culture temperature, and economic value. Pathogens and protection required also need to be considered. Economic cost is another essential factor for aquaculture industries, mostly related to the value of farmed fish.

Future fish vaccines against infectious pathogens, including viruses, bacteria, fungi, and parasites, in modern aquaculture industries should be cost-effective and environmentally friendly. They should allow for large-scale production and also be available and suitable for small fish farmers. Application of plant biotechnological techniques in fish vaccine development could satisfy these requirements for fish vaccination.

## Plant-Based Vaccine Development and Application

In molecular farming, recombinant proteins are produced in plants with the express intention to utilize the protein itself rather than any trait or capability it confers on the plant. Whole plants or plant cells/tissues cultured *in vitro* are used to express valuable recombinant proteins. This technique has been established as an economically viable alternative to mainstream production systems, such as microbes and mammalian cells cultivated in large-scale bioreactors (Clarke et al., 2013; Bock, 2015; Buyel et al., 2017; Dobrica et al., 2017; Castells-Graells and Lomonossoff, 2021). The initial attempts made to produce recombinant proteins in plants were in 1986 and 1989 (Barta et al., 1986; Hiatt et al., 1989). Utilization of plants for molecule production was initially adopted for biopharmaceutical proteins. Plant-produced human enzyme has been commercialized since 2012 (Rosales-Mendoza et al., 2017; van Eerde et al., 2020; Siriwattananon et al., 2021). A large number of pre-clinical and clinical studies of plant-made human and animal recombinant pharmaceutical proteins have proved the feasibility, efficacy, and safety of plant factories for vaccines (Takeyama et al., 2015).

As distinct from microbes or mammalian cell expression, a plant-based platform offers attractive advantages for recombinant subunit vaccines (Clarke et al., 2013; Rosales-Mendoza et al., 2017; Buyel, 2019): (1) It is an environmentally friendly vaccine production platform using free solar energy and capturing CO_2_, with low energy requirements and lacking greenhouse gas emissions reducing the cost of vaccines. (2) Plant-based systems can be used to produce vaccines for oral delivery which do not require sophisticated bioreactors and complex downstream processing, thereby becoming a more economical system than the conventional expression systems, e.g., *E. coli* (Avesani et al., 2010) and baculovirus-infected insect cells (Avesani et al., 2013). Hence, scaling-up of production is cheaper and the scale is not restricted by the size and number of available bioreactors (Daniell et al., 2015; Abiri et al., 2016). (3) Plant systems are safe. Plant-made subunit vaccines do not have the safety concerns of live vaccines (Su and Su, 2018). Moreover, undesirable or toxic components, such as bacterial endotoxins and hyperglycosylated proteins from yeast, have not been found in plant-derived systems, in contrast to mammalian-based production systems. (d) Plant-based systems possess a high capacity for biosynthesis, similar to naturally occurring systems, to perform posttranslational modification, such as glycosylation and complex folding and assembly, increasing vaccine immunogenicity (Kim et al., 2009; Guan et al., 2013; Lai and Qiang, 2013; Aboulata et al., 2014). On the other hand, plant-based platforms still possess disadvantages in uncertain efficiency of modifications including glycosylation, methylation, polymerization, quantity and quality of recombinant proteins, and dosage of vaccines in tissues (Erna et al., 2016).

Plenty of vaccine antigens, monoclonal antibodies (mAbs), and other biopharmaceuticals have been produced using three different plant biotechnology platforms: transient expression of transgenes in plants mediated by viral vectors, stable nuclear expression of transgenes in the nuclear genome of transgenic plants or cell cultures, and stable expression of transgenes in the plastid (chloroplast) genome of transplastomic plants (Daniell et al., 2009; Streatfield, 2010; Lössl and Waheed, 2011; Maliga and Bock, 2011; Clarke et al., 2013).

Each system has its advantages and limitations, and the choice of technique is largely dependent on the required production. Meanwhile, the regulatory issues of plant-made recombinant proteins and good manufacturing practice have also been well developed.

Various species of plants are available for plant molecular farming. Plastid genome engineering of edible plants has been utilized to produce a great many kinds of foreign proteins in lettuce, tomato, potato, cabbage, etc. (Ruf et al., 2001; Kanamoto et al., 2006; Cardi et al., 2010). A chloroplast expression system has been reported to be a promising production system for oral vaccines (Davoodi-Semiromi et al., 2010; Verma et al., 2010). Transgenic plants have been used to produce vaccines against viruses, bacteria, and parasites (Specht et al., 2010; Guan et al., 2013). Vaccine production in algae is another system used for transient expression and there are also some good examples of algal-produced biopharmaceuticals (Vidal-Meireles et al., 2017; Tabatabaei et al., 2019).

Microalgae offer health-promoting benefits as a nutritional supplement in feed meal for their digestibility, high contents of proteins, lipids, and essential nutrients, and potential immunogenicity (Charoonnart et al., 2018). Microalgae can be a viable protein production and oral delivery system to vaccinate fish. Genetic engineering technologies in microalgae offer the possibility of producing “functional feed additives” with no need for complex post-expression conditions, such as purification, cold chain, and injection (Kwon et al., 2019). *Chlamydomonas reinhardtii, Dunaliella salina*, cyanobacteria, and other microalgae have been processed to express antigen genes in chloroplasts for the prevention and control of infectious diseases (Ma et al., 2020b). Although several species of microalgae have been widely used in the aquaculture industry, many of them have not yet become established as an effective and mature genetic manipulation system.^3^

Vaccine development in plants has been covered in a number of reviews (Rybicki, 2014; Takeyama et al., 2015; Erna et al., 2016; Capell et al., 2020; Tuse et al., 2020). Human plant-based vaccines are not yet commercialized, while numerous subunit vaccine attempts are being performed in transgenic plants (Floss et al., 2007; He et al., 2008; Erna et al., 2016; Rosales-Mendoza et al., 2017). Hepatitis C virus E1E2 and its mutant E1E2ΔN6 have been produced in lettuce with a transient gene expression system and correctly processed and glycosylated. Oral vaccination with the lettuce induced vaccine-stimulated secretion of IgA (Immunoglobulin A) in mice (Clarke et al., 2017). This study provided the evidence that complex viral antigen could be produced, processed, and functionally modified in edible plants. Plant-made vaccines have been widely reported against rabies (Ashraf et al., 2005; Loza-Rubio et al., 2008; Roy et al., 2010; Loza-Rubio et al., 2012), porcine reproductive and respiratory syndrome virus, and porcine post-weaning diarrhea in piglets (Chen and Liu, 2011; Kolotilin et al., 2012). A tobacco-made Newcastle disease vaccine for poultry is the only plant-made vaccine approved by the United States Department of Agriculture so far (Hahn et al., 2007; Li et al., 2007; Yang et al., 2007; Joensuu et al., 2008; Gómez et al., 2009; Van Eck and Keen, 2009; Wu et al., 2009; Ma et al., 2020a). Plant-made veterinary vaccines have also been studied in mink, dogs, and cats (Dalsgaard et al., 1997; Molina et al., 2004, 2005; Rybicki, 2010).

Plant-based vaccines are ideal for oral administration. The manufacturing process is simple and there is no additional medical device needed for injection. Plant-based vaccines with high purity make vaccination more convenient and induce stronger immune responses. Vaccine antigens ingested by oral administration go through the gastric environment and to the intestine and are absorbed by M cells in the follicle-associated epithelium, inducing mucosal and systemic immune responses (Holmgren and Czerkinsky, 2005; Lamichhane et al., 2014; Tatsuhiko et al., 2014). Moreover, vaccines based on edible plants, such as lettuce, potato, tomato, corn, and rice, provide a needleless, convenient, and easy route of administration (Tacket, 2009). Lettuce is a potentially ideal plant base for oral vaccines and several human therapeutic proteins have been expressed at high levels in lettuce chloroplasts, e.g., oral vaccine from lettuce chloroplasts against dengue fever (Ruhlman et al., 2007, 2010; Davoodi-Semiromi et al., 2010; Boyhan and Daniell, 2011; Kanagaraj et al., 2011; Lakshmi et al., 2013; van Eerde et al., 2019).

Regulatory constraints are another obstacle for commercial GM plants worldwide, especially in Europe. Utilization of *Agrobacterium* for transformation or plant antigens, transgene containment and long-term stability under room temperature add to regulatory and production costs (Yusibov et al., 2011). Production in an open field system lowers production costs but adds significant regulatory costs for large-scale field research. Stable expression of virus subunits in chloroplasts does not use *Agrobacterium*, and without involving any plant pathogenic sequences are an ideal approach to minimize regulatory cost (Huebbers and Buyel, 2021). Meanwhile, avoiding production in seeds and using a greenhouse could effectively reduce costs in regulatory approval.

## The Potential for Plant-Made Fish Vaccines

For new fish vaccines, economic cost is one of the most important factors due to the nature of aquaculture with high farming cluster density and large scale. Plants provide a production platform for vaccines with high cost-effectiveness, efficiency, and safety (Ma et al., 2019). Despite having a higher monetary cost than antibiotics, plant-based vaccine production systems are still a cost-effective and promising approach offering efficient and safe vaccines to protect fish health and manage the sustainable development of fast-growing aquaculture globally. Diversity of fish species, type of fish diseases, characteristics of the vaccine target, timeline, scaling-up potential, resources, biosafety concerns, future commercial perspective, feeding habits of fish, and management difficulty also need to be considered prior to selection of a plant expression system for fish vaccine production.

The number of studies into the use of plants for the production of subunit vaccines for fish is still small but growing (Marsian et al., 2019; Su et al., 2021). Fish vaccines made in edible plants offer great potential for oral vaccination in aquaculture. A plant-generated recombinant subunit vaccine could also provide several antigen proteins simultaneously (Buyel, 2019). However, no plant-produced fish vaccine has been commercialized to date (Table 2). Therefore, it is extremely necessary with further research on fish vaccine production using plant biotechnology.

**Table 2:** Representative commercial fish vaccines and plant-produced fish vaccines under development.

Species	Disease or pathogen	Vaccine antigen	Production platform	Delivery route	Commercially available	Producer/reference
Salmonids	Infectious pancreatic necrosis virus (IPNV)	Inactivated virus	Inactivated	Intraperitoneal	Yes	Centrovet, Chile
Salmonids	Infectious hematopoietic necrosis virus (IHNV)	Recombinant G protein	DNA vaccine	Intramuscular	Yes	Aqua Health Ltd., Novartis, Canada
Salmonids	Salmon alphaviruses (SAV)	Inactivated virus	Inactivated	Intraperitoneal	Yes	Pharmaq AS, Norway Intervet-International BV, Netherlands
Koi carp	Koi herpes virus disease	Attenuated virus	Attenuated	Immersion/injection	Yes	KoVax Ltd., Jerusalem, Israel
Grass carp	Grass carp reovirus	Attenuated virus	Attenuated	Injection	Yes	Dahuanong, China
Channel catfish	*Edwardsiella ictaluri*	Attenuated bacteria	Attenuated	Immersion	Yes	Mississippi state university, United States
Grouper	Red grouper nervous necrosis virus (RGNNV)	Capsid protein	Yeast	Oral	No	Cho et al., 2017
Grouper	Orange-spotted grouper nervous necrosis virus (OSGNNV)	VLP	*E. coli*	Oral	No	Chien et al., 2018
Rainbow trout	IPNV; IHNV	IPNV VP2	RNA polymerase II system	Intraperitoneal	No	Guo et al., 2018
Salmonids	Cardiomyopathy syndrome (PMCV)	ORF1	*Nicotiana benthamiana*	Intraperitoneal	No	Su et al., 2021
Salmonids	Atlantic cod nervous necrosis virus (ACNNV)	VLP	*N. benthamiana*	Intramuscular	No	Marsian et al., 2019

Oral vaccination provides a non-stressful and energy-saving administration for both fish and farmers (Embregts and Forlenza, 2016). Vaccination *via* feeding appears to be an ideal method to provide protective immunity without extortionate cost of time and effort. Although taking into account the vaccination dosage and the cost of plant treatment including homogenization, drying, and briquetting, the use of edible plants for production of pathogen subunits decreases the need for expensive fermentation, purification, cold storage, transportation, and sterile delivery (Clarke et al., 2017), and oral recombinant plant-produced vaccine has the advantages of simplicity and safety. Thus, oral vaccine produced in edible crops offers unique cost advantages and antigen stability at room temperature.

By means of a plant expression system, VLPs assembled from viral capsid proteins mimicking natural viral tertiary structure (Rosenthal et al., 2014) have become an ideal advanced subunit vaccine candidate for fish vaccine. VLPs do not contain genetic material, avoiding the possibility of reversion mutations or pathogenic infection (Noad and Roy, 2003). However, VLPs can potentiate host immune responses by recognizable repetitive subunits triggering cellular and humoral responses (Keller et al., 2010). VLP vaccines have been licensed and commercialized in humans, such as Cervarix [human papillomavirus (HPV)], Merck’s Recombivax HB [Hepatitis B virus (HBV)], and Gardasil (HPV; Yusibov et al., 2011). There has been increasing interest in VLP vaccination of fish. Injection of NNV VLP vaccines produced by *E. coli*, yeast, baculovirus, and plant or cell-free self-assembled expression has been tested to elicit immune responses in fish, stimulating specific antibody secretion and triggering full-scale immune response (Lin et al., 2001; Liu et al., 2006; Lai et al., 2014; Luu et al., 2017). Oral VLP vaccines against grouper NNV can stimulate specific antibody production and provide over 50% protection against NNV challenge (Cho et al., 2017; Chien et al., 2018). Yeast expressed IPNV capsid protein VP2 subviral particle (SVP) vaccine in rainbow trout has been developed inducing specific antibody secretion, proving its immunogenicity (Dhar et al., 2010). IPNV VP2 protein recombinant vaccines against both IPNV and IHNV (infectious hematopoietic necrosis virus) have been shown to trigger IgM production against both pathogens (Guo et al., 2018). A plant-made VLP vaccine against piscine myocarditis virus (PMCV) in wild Atlantic salmon, the causative agent of cardiomyopathy syndrome, was produced in *Nicotiana benthamiana* by transient expression and reported recently by our laboratory. PMCV VLP vaccine provided limited protection against PMCV infection. Industrial scale fish vaccination trials have demonstrated the potential and also challenges encountered (Su et al., 2021). VLPs of Atlantic cod nervous necrosis virus (ACNNV) were successfully produced by transient expression in *N. benthamiana* and have been shown to provide efficient protection against virus challenge in sea bass (*D. labrax*; Marsian et al., 2019). The aforementioned research demonstrates the ability of VLP to be an ideal alternative vaccine candidate to conventional inactivated or attenuated live vaccines. Plant expression systems are a great option for VLP production with the special advantages of plant biotechnology. Thus, plant-made VLP vaccines are promising for fish viral diseases, raising hopes of their use in the near future.

Based on the experience from human vaccines produced in plants, the development of fish vaccines in edible crops or non-food crops for oral vaccination has great application potential. The few examples mentioned above strongly suggest that development of fish vaccine by plant biotechnology needs further research efforts to advance cost-effective fish health management and a sustainable aquaculture industry.

## Conclusion

Research on plant-based vaccines focusses mostly on increasing the amount and purity of antigen in transgenic plants to stimulate adequate immune responses. Targeting the most suitable subcellular compartment in plant cells is also of great importance for optimal quantity and quality of antigen. At present, production and application of plant-made vaccines still face several challenges, yet the promise and potential of better plant-based vaccines are attractive. Plant-based vaccine is an emerging type of vaccine and it is anticipated that regulatory approval will be granted eventually.

Because of the increase of both global population and food safety demand, aquatic health management is important worldwide. Plant-based fish vaccines could provide low-cost, high safety, and effective protection for farmed fish species. However, development of plant-made fish vaccine is still lagging behind efforts in producing plant-made vaccines for use in humans and non-aquatic animals. The present review has summarized the status of fish vaccine management and the application of plant genetic engineering in fish vaccine production. Plant-made recombinant protein vaccine using oral administration shows special advantages in production and vaccination. However, plant-made vaccine has not yet been approved for oral delivery.

In conclusion, for future fish vaccines, plant biotechnology provides a perfect option for production and vaccination. More research should be carried out to meet this urgent need.

## Author Contributions

HS and JC initiated and designed the overall concept and wrote the manuscript. All authors revised the manuscript, approved the final version, and approved it for publication.

## Conflict of Interest

The authors declare that the research was conducted in the absence of any commercial or financial relationships that could be construed as a potential conflict of interest.

## Publisher’s Note

All claims expressed in this article are solely those of the authors and do not necessarily represent those of their affiliated organizations, or those of the publisher, the editors and the reviewers. Any product that may be evaluated in this article, or claim that may be made by its manufacturer, is not guaranteed or endorsed by the publisher.

## Footnotes

^1^ http://www.fao.org

^2^ http://www.globalgoals.org/

^3^ https://thefishsite.com/articles/boost-for-algae-based-oral-vaccines; https://www.aquaculturenorthamerica.com/algae-based-vaccine-for-aquaculture-in-the-works-1804/; https://www.enabling-project.com/news-1/2020/2/11/algae-for-fish-vaccine
